# Classification of Frankfurters by FT-Raman Spectroscopy and Chemometric Methods

**DOI:** 10.3390/molecules191118980

**Published:** 2014-11-18

**Authors:** Náira da Silva Campos, Kamila de Sá Oliveira, Mariana Ramos Almeida, Rodrigo Stephani, Luiz Fernando Cappa de Oliveira

**Affiliations:** 1 Núcleo de Espectroscopia e Estrutura Molecular (NEEM), Departamento de Química, Universidade Federal de Juiz de Fora, Juiz de Fora, MG 36036-330, Brazil; E-Mails: nairacampos@ice.ufjf.br (N.S.C.); kamila@ice.ufjf.br (K.S.O.); rodrigo@gemacomtech.com (R.S.); 2 Instituto de Química, Universidade Estadual de Campinas, Campinas, SP 13084-971, Brazil; E-Mail: mariramosalmeida@gmail.com; 3 Gemacom Tech, Juiz de Fora, MG 36092-050, Brazil

**Keywords:** frankfurters, Raman spectroscopy, PLS-DA, quality control

## Abstract

Frankfurters are widely consumed all over the world, and the production requires a wide range of meat and non-meat ingredients. Due to these characteristics, frankfurters are products that can be easily adulterated with lower value meats, and the presence of undeclared species. Adulterations are often still difficult to detect, due the fact that the adulterant components are usually very similar to the authentic product. In this work, FT-Raman spectroscopy was employed as a rapid technique for assessing the quality of frankfurters. Based on information provided by the Raman spectra, a multivariate classification model was developed to identify the frankfurter type. The aim was to study three types of frankfurters (chicken, turkey and mixed meat) according to their Raman spectra, based on the fatty vibrational bands. Classification model was built using partial least square discriminant analysis (PLS-DA) and the performance model was evaluated in terms of sensitivity, specificity, accuracy, efficiency and Matthews’s correlation coefficient. The PLS-DA models give sensitivity and specificity values on the test set in the ranges of 88%–100%, showing good performance of the classification models. The work shows the Raman spectroscopy with chemometric tools can be used as an analytical tool in quality control of frankfurters.

## 1. Introduction

Nowadays, processed meat products are widely consumed all over the world. Among them, the most widely accepted by consumers are bologna, salami and frankfurters. The focus of this study was the frankfurter, which is considered to be a scalded or emulsified cooked product. This type of products is composed of muscle tissue, fat, water, salts and condiments that upon heat treatment acquire solid consistency [1]. In meat emulsions, the dispersed phase is formed by small globules and is composed of solid or liquid fat particles, the continuous phase consists of water, salts and proteins in suspension [2].

The production of processed meat products requires a wide range of meat and non-meat ingredients, each performing a specific function according to its property [3]. Due to these characteristics, the frankfurters are products that can be easily adulterated. However, the evaluation of the quality and authenticity in these products encompasses many issues, such as the fraudulent substitution of higher commercial value meats by lower value meats, the presence of undeclared species and the use of vegetable proteins, since they have a considerably lower price than muscle proteins [4]. Furthermore, the identification of adulteration is essential to the human diet in cases of moral, religious, cultural or health considerations [5]. Thus, rapid and reliable methods for evaluation of the kind and quality of frankfurters are necessary for implementation of food labeling regulations.

Several analytical techniques have been suggested for the identification of meat species, including different protein-based methods, such as high-performance liquid chromatography, electrophoretic techniques and enzyme-linked immunosorbent assays [4]. However, these techniques use expensive instruments, are destructive, time-consuming and generate toxic by-products. Although immunological methods are suitable, easy to perform and use a small quantity of reagents, the performance of such methods depends on the quality and availability of the antibody. So it is necessary to use other techniques that can overcome these disadvantages. Analytical tools based on spectroscopic methods are good alternatives, since they are non-destructive, rapid and low-cost techniques [4]. Recently, spectroscopic methods have been used to assess the quality and authenticity of meat products—Kamruzzaman* et al*. [6] used NIR hyperspectral imaging and multivariate image analysis to detect lamb meat adulteration; Alamprese and collaborators [7] detected minced beef adulteration by UV-vis, NIR and MIR spectroscopy; Kurniawati, Rohman and Triyana [8] made the analysis of lard in meatball using Fourier transform infrared spectroscopy and chemometrics; Foca* et al*. [9] classified pig fat samples from different subcutaneous layers by means of fast and non-destructive analytical techniques; De Marchi [10] used the near infrared spectroscopy to evaluate beef quality traits; Zhao* et al*., used NIR [11] and FT-IR [12] associated with multivariate modeling to detect adulterations in beef burgers. In all such investigations none of them addressed the content of lipids in frankfurters, which is the aim of this investigation.

The Raman data from a set of samples can be treated with multivariate analysis, where it can use all spectral range to extract information and to help in the interpretation of the data set. The development of analytical methodologies employing Raman spectroscopy and chemometric tools in the food area are demonstrated in the scientific literature [13]. When the goal is just to know the profile of the samples, performed an exploratory analysis using principal component analysis (PCA). If the samples profiles are known, a classification model can be constructed, and the chemometric tools as, linear discriminant analysis (LDA), soft independent modeling of class analogy (SIMCA) and partial least square discriminant analysis (PLS-DA) are most employed for this purpose. The determination of physical or chemical parameters of a set of samples can be obtained from information provided by Raman spectra and construction of multivariate calibration models.

In this work, Raman spectroscopy was used to discriminate frankfurters from different sources; this technique provides molecular-specific information, is not destructive, does not require sample preparation (thus saving on time and reagents), can determine more than one component at a time, and is free from water interference in the analysis [14,15]. In this study, a screening method was developed using Raman spectroscopy and classification chemometric tools. The aim was to identify and classify three types of frankfurters (chicken, turkey and mixed) on the basis of their Raman spectra. Multivariate classification model was built using PLS-DA. The performance model was evaluated in terms of sensitivity, specificity, accuracy, efficiency, and Matthews’s correlation coefficient for both a training and validation set.

## 2. Results and Discussion

**Figure 1:**
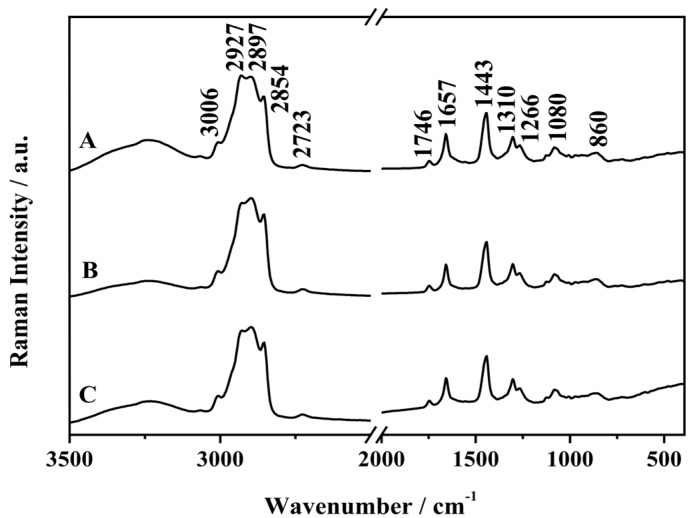
Average FT-Raman spectra of the frankfurters samples: (A) Turkey, (B) Mixture meat and (C) Chicken.

The Raman spectra obtained for the three different types of frankfurters are given in Figure 1 and the main vibrational bands are listed in Table 1, with their respective tentative assignments based on comparisons with previously published data. From the observed bands, it is possible to determine the chemical composition of the several types of frankfurters and it is also possible to see a big difference in intensity between these three types of samples. As said before, Brazilian law for frankfurters [16] establishes that the maximum levels of carbohydrates and total fats are, respectively, 7% (w/w) and 30% (w/w), and the minimum protein content is 12% (w/w).

**Table 1:** Tentative assignment of the Raman spectrum of frankfurters, based on similar systems described on literature [5,14,17,18,19,20].

Wavenumber (cm^−1^)	Tentative Assignment
3006	ν_sym_(=C–H)
2927	ν_ass_(CH_2_)
2897	ν_sym_(CH_3_)
2854	ν_sym_(CH_2_)
2723	ν(C–H)
1746	ν(C=O)_ester_
1657	ν(C=O) Amide I; ν(C=C)
1443	δ(CH_2_)
1301	τ(CH_2_) Amide III
1266	τ(CH_3_) + ν (C–N) Amide III
1080	ν(C-O) + ν(C–C) + δ(C–O–H)
860	Tyr

In the FT-Raman spectra, there is a band at 3006 cm^−1^ assigned to the symmetrical stretching of the (=C–H) group from unsaturated fatty acids present in the sample [18]. The band at 2930 cm^−1^ can be assigned to the (CH_2_) asymmetric stretching, and is related to proteins and carbohydrates vibrational modes.

In the regions of 2900 cm^−1^ and 2850 cm^−1^ was possible to observe two bands in all three types of related samples, respectively assigned to the symmetrical stretching of the CH_2_ and to the symmetrical stretching of CH_3_, which are characteristic vibrational modes of fats present in the samples. There is a difference in the intensity of these Raman bands, justified by the difference in fat content in all the samples. The mixed frankfurter has a fat content greater than both the chicken and the turkey frankfurters, where the latter has the lowest levels.

The spectra also show a band with moderate intensity in the region of 1750 cm^−1^, which can be assigned to the (C=O) stretching vibrational mode of fatty acids present in the samples, then being indicative of the fatty compounds and used as finger print for such family of compounds.

Raman spectroscopy provides information about proteins present in samples of frankfurter through the amide I (1645–1685 cm^−1^) and amide III (1200–1350 cm^−1^) vibrational modes. In the 1660 cm^−1^ region, it can be seen a band with a medium intensity in all the spectra, which is characterized by amide I mode, referring to (C=O) stretching of proteins. This region has also a contribution of the (C=C) bond stretching of the unsaturated fatty acids present in the samples. In the region of 1440 cm^−1^, it was observed a band, which has been assigned to the (CH_2_) scissoring mode related to the vibrational modes of lipids [19].

The region between 1200 and 800 cm^−1^ is dominated by bands assigned to vibrational modes of carbohydrates; the main vibrational modes are the C–C and C–O stretching and O–H deformation modes [5,14,17,18,20]. By comparing the Raman spectra of the frankfurters was not possible to observe visual differences in spectral profiles to classify the frankfurter types; thus, the use of chemometric tools was necessary for the construction of classification models.

Exploratory analysis employing PCA was performed to verify the behavior of the samples. PCA transforms complex data so that the most important and relevant information becomes easier to be seen while preserving as much as possible the original information. This is done by using a linear combination of original variables that generates these data, taking into account the variance associated with them [14]. The data set was pre-processed using the following sequence: first derivative, vector normalization and mean center; the numbers of principal components were selected on the basis of RMSECV (root mean square error of cross-validation). In this study were used four principal components representing 97.39% of the total variance.

The use of pre-processing had the goal to remove the effects of baseline spectrum, which varies depending of the experimental conditions, and to normalize the data. The first derivative measures the slope of the curve and is not affected by the effects caused by the change from baseline, but may increase the noise. In the vector normalization the mean-centered spectra are divided by the square root of the sum of the mean-centered intensities squared. Mean center consists of subtracting from each variable the mean value of the variables [21].

**Figure 2:**
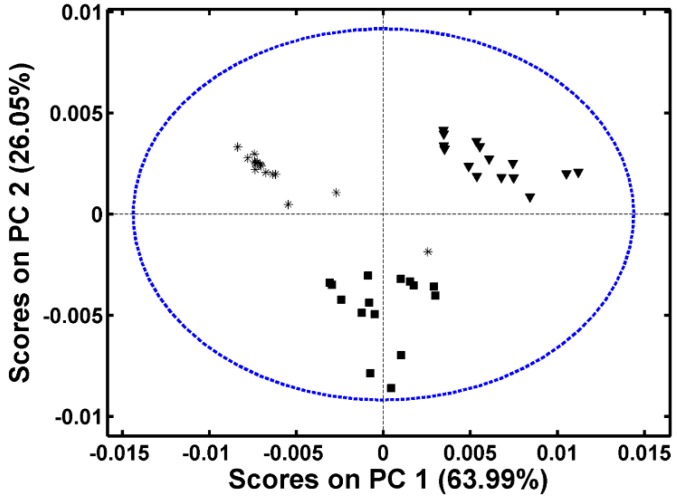
Plot of the scores of PC1 × PC2 of chicken frankfurter (■), turkey frankfurter (▼) and mixture meat frankfurter (_*_).

The projection of the samples onto the first two principal components, Figure 2, allows the observation of the samples distribution and the analysis of their grouping, corresponding to different classes of frankfurters. The ellipse in Figure 2 defines the Hotelling’s T^2^ limit and is used to identify outliers. The first principal component separates turkey frankfurters samples from the mixed frankfurters samples, while the second principal component separates the samples of chicken from the others. It is observed that a mixed frankfurter sample is close to the chicken frankfurter group. Once the mixed frankfurter is a mixture of different meats, the amount of chicken meat in this sample must be higher than in the other.

**Figure 3:**
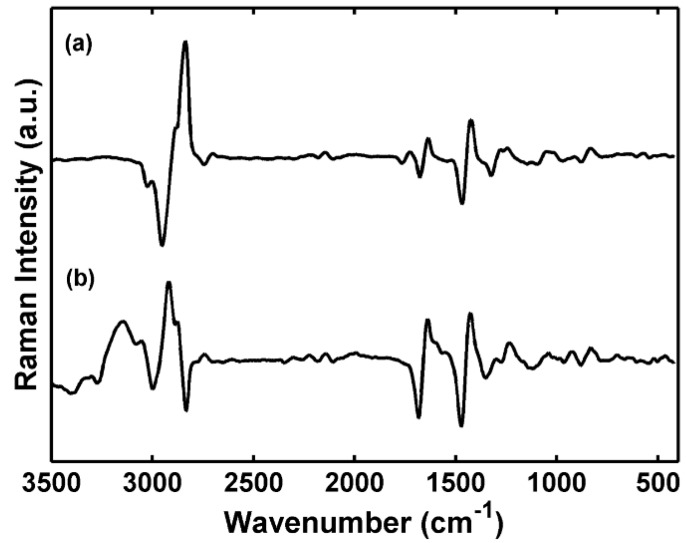
Plot of the loadings of (**a**) PC1, (**b**) PC2* versus* wavenumber (variables) for the PCA model.

The loadings analyses of principal components provide spectral information about the difference between the classes of frankfurters. The graph of loadings of the first principal component, Figure 3, shows the Raman spectral regions that are responsible for the separation of the turkey and mixed samples. The region at 2900 cm^−1^ has a high loading value; this region is characteristic of fat. So, the separation of the samples in score graphs is based on the fat amount of the samples; as it can be seen in the PC1 in Figure 2, the fat concentration increases from turkey and chicken frankfurters samples to mixed meat frankfurters samples. The PC2 loading shows the contribution of Raman band at 3200 cm^−1^, which is attributed to OH stretching and is related to moisture content in the samples. Analyzing the score graph (Figure 2) it can infer that moisture content in the chicken frankfurter is different from the other frankfurter samples. The regions at 1750 and 1450 cm^−1^ also contribute to differentiation of frankfurter samples, and are assigned to the (C=O) stretching vibrational mode and (CH_2_) scissoring mode related to the vibrational modes of lipids, respectively. Although PCA provides a satisfactory result in relation to the frankfurter type, this is an unsupervised method and cannot be used in terms of classification. In this case, discriminant analysis using PLS-DA is more appropriate, since classification is the main goal.

To build a classification model, the samples were separated into two data sets: training and validation set. The training set is used to build the rule of classification and the test set to the validation model (external validation). The training set was composed of 38 samples, 16 chicken frankfurter samples, 13 mixed frankfurter samples and 9 turkey frankfurter samples. The test set consisted of 19 samples, 2 chicken frankfurter samples, 8 mixed frankfurter samples and 9 turkey frankfurter samples. Training samples must be representative of the experimental space, because they are used to build classification rules. In this work the training samples (calibration set) were selected using the Kennard-Stone algorithm, [22], which starts by selecting the first sample with the highest distance from the sample mean. The second sample will be selected to present the greatest distance from the first selected sample. The next sample to be selected will present greater distance from the last selected sample, and so on until the desired number of samples. Thus, is made the selection of samples of greater variability. The partial least square discriminant analysis (PLS-DA) was applied to the training set and then the developed models were validated using test set samples.

**Figure 4:**
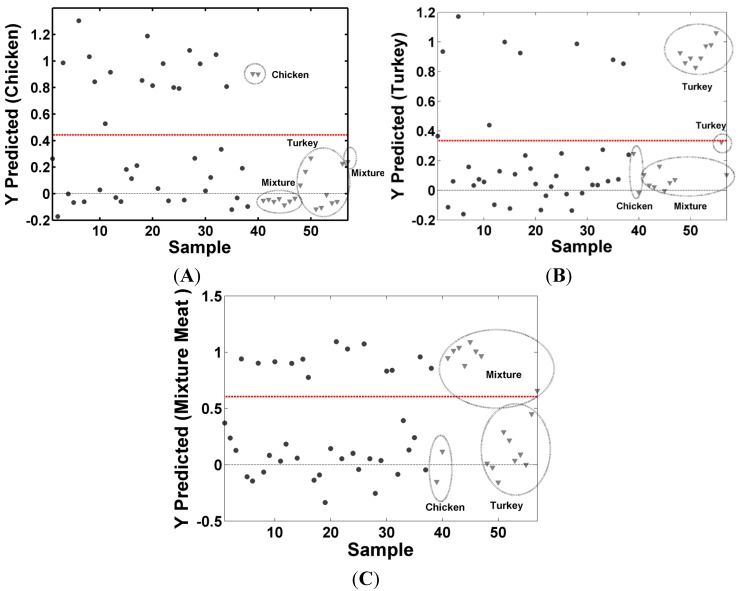
Results of the training and test set of the PLS-DA model: ● (training samples), ▼ (test samples); (**A**) chicken frankfurters; (**B**) turkey frankfurters and (**C**) Mixture meat frankfurters.

The training and test set were pre-processed using first derivative, vector normalization and mean center for **X** matrix and mean center for **Y** matrix. The choice of the number of the latent variables was made by cross-validation using the venetian-blinds method with number of data splits equal to 5. Based on the lowest value of the RMSECV, four latent variables were chosen to build the classification model, representing 98% of the variance explained in the **X** and 85% of the variance in the **Y** matrix. After choosing the number of latent variables, the performance of the classification model was evaluated by internal validation (cross-validation) and external validation (new samples) using the test set. The class predictions calculated by the PLS-DA models for the training and test sets are shown in Figure 4; for the validation set, the mean value of RMSEP for the classes were 0.15, this value shows a model ability to predict new samples and is an estimate of the accuracy of the model. The values of RMSEC and RMSEP show a good agreement, indicating that the RMSEC value is a good estimative of the standard error of prediction observed for the test set. The class a sample belongs to is determined by a value between 0 and 1. The decision rule for classification is defined by dashed horizontal line, shows in Figure 4, if the value is above line, assign it to class chicken frankfurters in Figure 4A, turkey frankfurters in Figure 4B and mixture meat frankfurter in Figure 4C; if the value is below line, the sample does not belong to the class in question.

The classifications performance of the classification models were evaluated by sensitivity, specificity, accuracy, efficiency, and Matthews’s correlation coefficient. The results obtained for the training, cross-validations and test set samples are summarized in Table 2. The PLS-DA models give sensitivity and specificity values on the test set in the ranges between 88% and 100%, showing good performance of the classification models. Accuracy and efficiency were also evaluated; the accuracy shows the proportion of samples identified correctly, which on the tests sets were equal to or higher than 90%. The efficiency combines all of the information carried by sensitivity and specificity; when a method is very sensitive to positive, it generates many false positives, and vice versa. For the test sets, the efficiency values were higher than 90%. The Matthew’s coefficient value for the tests sets were 0.9 and one, a value that can classify the methodology as having a good performance.

**Table 2:** Classification parameters obtained for PLS-DA. 1. Chicken frankfurters; 2. Turkey frankfurters; 3. Mixture meat frankfurters.

	Training Set						Validation Set		
	Calibration			Cross-Validation			Test		
	**1**	**2**	**3**	**1**	**2**	**3**	**1**	**2**	**3**
**Sensitivity (%)**	93.8	88.9	100	87.5	77.8	92.3	100	88.9	100
**Specificity (%)**	100	96.6	100	95.5	93.1	100	100	100	100
**Accuracy (%)**	97.4	94.7	100	92.1	89.5	97.4	100	94.7	100
**Efficiency (%)**	96.9	92.7	100	91.5	85.4	96.2	100	94.4	100
**Matthew’s Correlation**	0.95	0.85	1.00	0.84	0.71	0.94	1.00	0.90	1.00

The best results were obtained for the mixture meat frankfurters samples, all samples were correctly classified in the training set and the test set. These samples have higher fat content, thereby, have different spectral characteristics from the other samples. The classification models for chicken and turkey frankfurters showed small errors for the training samples, which can be justified due to the spectral similarities between the two types of samples. In general, the behavior of these models can be considered very satisfactory. In all the models the results obtained with training as test were comparable, with small differences in the corresponding parameters, this is an indicative that over-fitting did not occur and a high reliability of the models.

## 3. Experimental Section

### 3.1. Samples

Fifty-seven samples of two different brands of frankfurters were obtained from commercial market in Juiz de Fora city, Minas Gerais, Brazil. Three types of samples were studied, including chicken, turkey and mixed meat frankfurters. The Raman spectra were obtained without the use of any physical and/or chemical pretreatments. It is important to note that the only information about composition of the frankfurter sausages is the one contained in the label of each one of the studied samples; however, Brazilian law for frankfurters [16] establishes that the maximum content for carbohydrates and total fats are 7% (w/w) and 30% (w/w), respectively, and the minimum protein content is 12% (w/w). In the investigated samples the one made by turkey meat is announced as low fat content, if compared to the other ones, whereas the mixed meat frankfurter is made by a mixture of pork, cow and chicken meats, once again based on the label information.

### 3.2. FT-Raman Spectra Acquisition

The Raman spectra of the samples were collected on an RFS 100 FT-Raman Bruker spectrometer equipped with a Ge detector using liquid nitrogen as the coolant and excited with a 1064 nm beam from a Nd: YAG laser. The samples were cut into small pieces, placed on a glass plate, without any pretreatment, and taken for analysis in the spectrometer; the laser light, with a power of 150 mW in the sample, was focused on the sample, and the scattered radiation was collected at 180° in a backscattering geometry; it is straightforward to say that the best focus laser spot is about 0.5 mm. For each spectrum, an average of 1000 scans (*ca.* 15 min) was collected with a resolution of 8 cm^−1^ over the range between 3500 and 400 cm^−1^. These parameters were selected in order to obtain the best signal-to-noise ratio while the physical and chemical integrity of the samples was maintained; this was achieved by repeating each one of the spectra and observing the possible changes in position and intensity over all the spectrum bands. A Blackman–Harris-3 term was used as the apodization function, and a zero-filling factor of two was employed. The software OPUS 6.0 (Bruker Optik, Ettlingen, Germany) was used for Raman data acquisition.

### 3.3. Chemometric Analysis

To develop the classification model the Raman spectra were manipulated using MATLAB software Version 7.10 (R2010a).

A problem in Raman spectroscopy is the presence of effects due to several events, such as particle size, morphological differences, detector artifacts and experimental condition dependence, which should be eliminated before use of chemometric tools, since they can alter the intensity of the spectra. Then, the Raman spectra were preprocessed using baseline correction to minimize the effect of the fluctuations on the baseline and normalization. The first derivative done by Savitzky-Golay algorithm with second-order polynomial and window with 15 points was applied for baseline correction and normalization procedure was made by vector normalization, where the sum of all intensities values squared equals one. The normalization is used to scale the spectra within a similar range and allows a comparison between the heterogeneous samples.

Following, principal component analysis (PCA) was used for exploratory analyses and feature reduction, and the components number was choose based on the explained variance. The loadings analysis allows evaluating the contribution of the Raman bands for separation of the samples.

Then, PLS-DA was applied for classification purposes; for construction of the PLS-DA models, it is necessary a matrix **X** that contains the Raman spectra and a **Y** matrix, that is related to the interest property, in this case the classes of samples. Three binary classification model were constructed, for the first model **Y** was created with 1 for chicken frankfurter and 0 for mixed and turkey frankfurters; for the second model, a new **Y** matrix was created with 1 for mixed frankfurter and 0 for chicken and turkey frankfurters and for the third model, Y matrix with 1 for turkey frankfurter and 0 for mixed and chicken frankfurters.

To build the classification model, the data set was divided in training and test sets. The samples were divided using Kennard-Stone algorithm [22]. The choice of the samples of the training set is based on the distances between the samples; thus, samples with higher variability are selected, creating a representative set. For the training set 38 samples was selected. The latent variables number for PLS-DA model was chosen by the criterion of lowest prediction error cross-validation, this criterion reduces the risk of over fitting. The prediction ability of the model was evaluated by studying the root mean square error of calibration (RMSEC) and cross-validation (RMSECV) of the training data. The performance of the model was estimated in terms of the root mean square error of prediction (RMSEP) using test set (external validation) with 19 samples, which was not included in the model construction.

The threshold between classes was estimated by Bayesian methods using the training set. The threshold value for the class separation of each model was selected based on the lowest number of false positives and false negatives, these numbers should be minimized for future predictions [21]. The evaluation of classification performance parameters was performed by Confusion Table parameters, such as sensitivity, specificity, accuracy, efficiency were calculated and used to evaluate the performance of the model.

The sensitivity is the ability of the model to correctly classify the frankfurter class. It is calculated according to [23]:

(1) $$Sensitivity=\frac{tp}{tp+fn}$$

where *fn* are false negative samples and *tp* are true positive samples.

The specificity is the capacity of the model to correctly identify the samples that don’t belong to the modeled class [23]:

(2) $$Specificity=\frac{tn}{tn+fp}$$

where *fp* are false positives samples and *tn* are true negatives samples

The accuracy is the proportion of correct classification, independent of the class [23]:

(3) $$Accuracy=\frac{tp+tn}{total\;samples}$$

Efficiency and Matthew’s correlation coefficient can be used to provide a single measure of model performance. The efficiency combines all information carried by the sensitivity and specificity [23].

(4) $$Efficiency=\frac{Sensitivity+Specificity}{2}$$

Matthew’s correlation coefficient summarizes the quality of the classification model in a single numerical value, which can be used even if the classes have different sizes [23].

(5) $$Matthew^{\prime }s\;coefficient=\frac{\left(tp\times tn\right)-\left(fp\times fn\right)}{\sqrt{\left(tp+fp\right)\times \left(tp+fn\right)\times \left(tn+fp\right)\times \left(tn+fn\right)}}$$

This expression returns a value between −1 and +1, where a value of +1 represents a perfect classification, 0 an erroneous classification and −1 an inverse classification.

## 4. Conclusions

Raman spectroscopy has proven to be a useful technique to study frankfurters samples. Furthermore, this type of spectroscopy does not require any treatment of samples, generates no toxic residues and the analysis time is short. The use of Raman spectroscopy and PLS-DA allows the distinguishing between the frankfurters types in terms of fatty content, mainly based on the model where the bands that are the most relevant are the ones assigned to the presence of fat. The methodology proposed can be used as screening test to control the quality of products. The results show that the Raman spectroscopy together with PLS-DA has the potential to be an alternative for the standard procedures used for frankfurter analysis, as well as to quality control in terms of fatty content and in terms of the type of used meat, since several different meats have very different fatty contents, for instance the ones analyzed here (chicken, turkey and mixed meats). At last, we have done an investigation based on the Raman bands assigned to fatty compounds, present in the three types of frankfurters we can find in market. We are not concerned if manufacturers are really using or not such types of meat to produce the frankfurters; in fact, the main focus is the separation and qualification based on the spectroscopic features we are seeing. This separation, based on the bands from lipids, can be used to identify and qualify the frankfurter samples.
